# Botulinum Toxin for the Treatment of Myofascial Pain Syndromes Involving the Neck and Back: A Review from a Clinical Perspective

**DOI:** 10.1155/2013/381459

**Published:** 2013-02-19

**Authors:** José M. Climent, Ta-Shen Kuan, Pedro Fenollosa, Francisco Martin-del-Rosario

**Affiliations:** ^1^Physical and Rehabilitation Medicine Department, Alicante University General Hospital, C/Pintor Baeza s/n, 03010 Alicante, Spain; ^2^Department of Physical Medicine and Rehabilitation, National Cheng Kung University Hospital, College of Medicine, National Cheng Kung University, Tainan 704, Taiwan; ^3^Pain Clinic, La Fe Hospital, 46026 Valencia, Spain; ^4^Physical and Rehabilitation Medicine Department, Gran Canaria Insular Hospital, Avenida Marítima del Sur, 35006 Las Palmas de Gran Canaria, Spain

## Abstract

*Introduction*. Botulinum toxin inhibits acetylcholine (ACh) release and probably blocks some nociceptive neurotransmitters. It has been suggested that the development of myofascial trigger points (MTrP) is related to an excess release of ACh to increase the number of sensitized nociceptors. Although the use of botulinum toxin to treat myofascial pain syndrome (MPS) has been investigated in many clinical trials, the results are contradictory. The objective of this paper is to identify sources of variability that could explain these differences in the results. *Material and Methods*. We performed a content analysis of the clinical trials and systematic reviews of MPS. *Results and Discussion*. Sources of differences in studies were found in the diagnostic and selection criteria, the muscles injected, the injection technique, the number of trigger points injected, the dosage of botulinum toxin used, treatments for control group, outcome measures, and duration of followup. The contradictory results regarding the efficacy of botulinum toxin A in MPS associated with neck and back pain do not allow this treatment to be recommended or rejected. There is evidence that botulinum toxin could be useful in specific myofascial regions such as piriformis syndrome. It could also be useful in patients with refractory MPS that has not responded to other myofascial injection therapies.

## 1. Introduction

Myofascial pain syndrome (MPS) is defined as a regional pain disorder of muscular origin characterised by the existence of trigger points within muscles. The myofascial trigger point (MTrP) is, in turn, defined as a palpable and hyperirritable nodule located in a taut band of muscle. Stimulation of this point produces two characteristic phenomena: referred pain and sudden contractions of the taut band, called the local twitch response (LTR). Active MTrPs produce pain, and sometimes referred pain, spontaneously. Latent MTrPs produce referred pain as a response to pressure, but not spontaneously.

A current hypothesis is that the disorder underlying MPS is related to inappropriate activity of acetylcholine (ACh) at the neuromuscular junction, which produces a sustained contraction of the sarcomere. The ACh-related effects are relevant to the development of the taut band. This activity leads to an increase in local energy demand or energy crisis [1]. Local muscle pain occurs because of the release of substances from damaged muscle, and from the extracellular fluid around the TrP, such as protons (H+) on acid-sensing ion channels [2], which occurs in ischemia and in exercise [3]. Under these metabolic conditions, sensitising amines that stimulate the nociceptors may be released, giving rise to pain. There are therefore two phenomena that better define MTrPs: altered ACh activity and nociceptive stimulation [4, 5].

The inappropriate activity at the motor endplate has been studied from an electrophysiological perspective. First, the existence of spontaneous electrical activity (SEA), characterised by continuous low-amplitude action potentials and spikes, was demonstrated in the active MTrP. Excessive ACh activity at the TrP (the muscle endplate) is inferred from the electrophysiologic activity (endplate noise and SEA) [6, 7]. In the other hand, two studies performed on the trapezius muscle identified a significant rise in the concentration of substance P, calcitonin gene-related peptide (CGRP), and other nociceptive neurotransmitters in the biochemical milieu of active MTrP [8, 9].

Another factor that plays a determining role in MPS is the sensitization phenomenon. Persistent peripheral muscle nociceptor activation is converted into a permanent stimulus that facilitates pain neurotransmission. This is due both to a local increase in the number of nociceptors and to the opening of silent multisegment spinal cord circuits [10]. This cytokine activation is critical for central sensitization and glial activation. Glial activation is also important, creating and maintaining enhanced pain states. When glia become activated, pain is amplified [11].

In summary, the disorder underlying MPS is considered to be inappropriate ACh activity at the endplate, producing an energy crisis that favours nociceptive neurotransmitter release. The altered ACh produces active phenomena (taut band), and the nociceptive neurotransmitters initiate the cascade of pain neurotransmission or sensory phenomena: local pain and referred pain.

Botulinum toxin has been used for decades in the treatment of disorders characterised by muscle hyperactivity, such as spasticity or dystonia [12]. Its analgesic potential was observed when, in addition to decreasing muscle hyperactivity, it was found to improve the pain in patients with dystonia [13].

*Clostridium botulinum* produces seven neurotoxins (designated by the letters A to G). Their best known action is the blockade of exocytosis of the presynaptic vesicles of ACh at the endplate. Two of these neurotoxins, botulinum toxin A (BTA) and botulinum toxin B, are available as biological therapeutic agents and may frequently be used for the treatment of certain conditions involving muscle hyperactivity [14]. BTA is a 150 kilodalton protein formed of a light chain (50 kDa, amino acids 1–448) and a heavy chain (100 kDa, amino acids 449–1280) joined by a disulphide bridge [15].

Botulinum toxin blocks neurotransmission at the neuromuscular junction. Several transport proteins participate in the process by which ACh is released; these proteins aggregate to form the SNARE complex (Soluble NSF (N-Ethylmaleimide-Sensitive Factor) Attachment Protein Receptor [16], responsible for fusion of the vesicles of ACh with the membrane and the subsequent release of the neurotransmitter. The heavy chain of the toxin has a high affinity for the membrane receptors and, once bound, BTA undergoes endocytosis. The light chain is released within the cell, where it acts as a zinc-dependent endoprotease [16–18]. After cleavage of one of the proteins of the SNARE complex by BTA, the complex does not form and ACh is not released.

### 1.1. Mechanism of Action for Pain Relief

A number of possible mechanisms of action that could explain the antinociceptive effects of BTA have been formulated and investigated [19].

#### 1.1.1. Reduction of MPS-Linked Hyperactivity

In myofascial syndrome it is believed that the excessive ACh production is responsible for the characteristic SEA of MTrPs, detectable on electromyography (EMG). The injection of 10 U of onabotulinumtoxinA (Botox) in the area of the dysfunctional motor endplate was found to reduce SEA in experimental animals [20].

#### 1.1.2. Direct Antinociceptive Effect

It has been shown that BTA directly inhibits the release of pain mediators such as substance P, bradykinin, CGRP, and glutamate [21, 22].

#### 1.1.3. Reduction the Sensitization Phenomenon

Nociceptive sensitization involves an increase in the concentration of substances that facilitate nociceptive neurotransmission, such as substance P, CGRP, and glutamate, both at the peripheral nociceptors and in the posterior horn of the spinal cord. Blockade of the release of these substances peripherally interrupts the first step of sensitization: the accumulation of nociceptive neurotransmitters at the free nerve endings. BTA is therefore considered to be more effective when sensitization phenomena exist [23, 24] than when they are absent, such as, for example, in acute pain.

As a result, BTA has recognised mechanisms based on experimental studies that would enable it to act on three critical aspects of MPS: excess Ach release, local nociception, and sensitization phenomena. These are the three reasons that have driven research into the analgesic potential of BTA in the myofascial pain syndrome and in other pain syndromes.

### 1.2. Clinical Experience

The first clinical trial on the treatment of MPS with BTA was published in 1994, and the results were promising [25]. Since that time, almost two decades have passed and many more studies have been published; however, the results have been contradictory, with reports both of nonsuperiority and of superiority of BTA compared with other treatments. A number of systematic reviews have also been published, including meta-analyses, though their conclusions have also been inconsistent.

Given this lack of uniformity or, at least, of similarity between the results and conclusions of those articles, the proposal of the present review is to analyse the publications from a clinical perspective to search for clues that could explain the differences. This is not a systematic review, but rather a critical analysis that aims to examine certain factors that could improve our understanding of the data published to date and of the discrepancies between those data.

The objective of this paper was therefore to conduct a qualitative analysis of the possible sources of variability between the different trials and reviews of the use of BTA for the treatment of myofascial pain syndrome.

## 2. Material and Methods

Literature searches were performed in the PubMed database using the following key words: “botulinum toxin” “myofascial pain”, and “botulinum toxin” “trigger point”. The results obtained were filtered to select those clinical trials and systematic reviews that referred to MPS associated with neck or back pain.

The clinical trials were studied from a qualitative point of view, recording specific data on the following aspects: diagnostic criteria, muscles injected, injection procedure, treatment for control group, and outcome measures. All these categories were studied using content analysis to search for possible sources of variability.

The conclusions of the reviews were studied and, after qualitative analysis of each category, a panel of discordant points was drawn up in order to highlight the sources of variability and suggest ways to achieve uniformity.

## 3. Results and Discussion

Nineteen clinical trials [26–44] and 15 systematic reviews [45–59] satisfied the selection criteria. Tables showing variables studied in every trial has been published previously [48, 50, 51, 58, 59].

Below we describe the possible sources of variability according to the established categories.

### 3.1. Diagnostic Criteria and Topography of Pain

Given that there is no definitive consensus on the diagnostic criteria of MPS, it is not surprising that studies on the use of BTA for the treatment of MTrPs apply different criteria. There are expert recommendations that propose a series of clinical criteria to make the diagnosis [1, 60]: focal spot muscle tenderness, a taut band running the length of the muscle, pressure-elicited referred pain pattern, pain recognition sign, LTR to stimulation of the muscle by pressure or needling, and other less specific signs, such as regional weakness without atrophy and mild limitation of the range of movement.

Although efforts are being made to establish diagnostic imaging for MPS, particularly with elastography techniques [61], we have still not reached the point at which it is possible to make the diagnosis based on these methods. The combination of signs most widely used in the literature to establish a diagnosis of MPS is the following: tender spot in a taut band, patient pain recognition on tender spot palpation, predicted pain referral on spot palpation, LTR and limited range of movement [62]. Usually, from a treatment point of view, only three criteria are necessary as well as sufficient: taut band, tenderness, and reproduction of pain.

However, the diagnostic criteria used were not detailed in the majority of studies, and it was simply stated that the patients suffered myofascial pain [28, 32, 34, 36]. One study did define two specific criteria to select the MTrPs suitable for injection: the pain recognition sign and pain elimination by compression [37]. Although it is possible to detect percentage improvements in the pain with compression therapy [63], the abolition of pain by compression is not usually considered a diagnostic criterion. Finally, a combination of criteria similar to those previously defined by Simons was detailed in two studies [33, 35].

There was also very marked variability between the trials with regard to the concept of pain topography. It must be realised that although MPS has traditionally been defined as specific to each muscle, it is actually a form of regional or widespread pain [1] and MTrPs can usually be detected in several muscles simultaneously [64]. The majority of studies adopt this approach, selecting patients with headache and neck pain [29, 30], pain in the neck and/or shoulder [34–37], shoulder and arm [33], neck and upper back [32, 44], neck, shoulder, hip or back [28, 36], chronic low back pain [41], or piriformis syndrome [42]. This spectrum of regional pain makes variability unavoidable, both between different studies and within individual studies. One could argue that the trigger point is the same no matter where it occurs and therefore the response to BTA should be the same in any muscle. However, there may be a factor influencing the answer is different depending on the muscles addressed as follows from the fact that some reports for especific muscle syndromes, such as piriformis, are favorable. One factor may be that most studies do not address the entire functional muscle unit involved by trigger points, and therefore, treatment has been incomplete in many studies, affecting the outcome.

An example of how the selection criteria can group together apparently similar but in reality profoundly different samples can be seen if we analyse two of the most detailed studies that have been published to date. In the study by Ferrante et al. there were 142 patients with myofascial pain of the neck or shoulder [37]. The trial by Göbel et al. included 144 patients with myofascial pain of the neck or shoulder [34]. These studies represent the two largest series published in this field. Both used one arm with BTA and another with normal saline. The two series differed in their results and conclusions: the study by Ferrante showed no significant differences between the arm treated with BTA and the one receiving normal saline. The series by Göbel, on the other hand, did detect significant differences in favour of BTA. Up to here the story is perfectly coherent. However, detailed reading of the selection and inclusion criteria of the two studies reveals certain key details. Ferrante applied the following exclusion criteria: (1) a total of more than five active trigger points, (2) more than two trigger points in the trapezius muscle on either side of the body, and (3) more than one trigger point in any other single surface muscle on either side of the body. In contrast, Göbel only included patients if they had at least 10 trigger points. These criteria mean that none of the patients selected by Göbel would have been included in the study by Ferrante as they all had more than 5 MTrPs. Likewise, none of the patients recruited to the study by Ferrante would have been selected by Göbel, as they all had fewer than 10 MTrPs. As a result, two of the largest and most powerful studies published to date were conducted with patients with MPS of such different characteristics that satisfaction of the conditions for recruitment to one trial would mean exclusion from the other.

Another aspect that was not detailed in the studies was whether the syndromes detected in the patients were primary or secondary. Primary MPS is an independent medical entity, whereas secondary MPS develops in association with other diseases, such as vertebral disc disease, nerve root disease, osteoarthritis, facet joint disease, cervical whiplash or after a muscle lesion [5, 64–66]. These clinical conditions could have affected the final results of the trials; however, they could be helpful to identify subgroups of patients with more or less favourable results.

In summary, the following sources of variability in the diagnosis were detected: lack of uniformity in the criteria used to diagnose MPS, variability in the regional pain topographies included in the studies and in the minimum and maximum numbers of MTrPs in any given patient in order to satisfy the recruitment criteria, and a lack of information about the clinical characteristics of the MPS and possible associated abnormalities.

### 3.2. Muscles Injected

In view of the diagnostic and topographic variability, we cannot expect greater uniformity in the muscles or muscle groups injected. One aspect that makes it difficult to reproduce certain studies is that the specific muscles injected are not identified in the study reports [30, 31, 34, 37]. Other studies sometimes do not give a complete description; for example, Lew et al. report that the trapezius, levator scapulae, splenius capitis, and other posterior neck muscles were injected [32]. Some studies are more specific, such as the one by Ojala et al., in which it was stated that the injections were made into the trapezius, levator scapulae and infraspinatus [35], and the study by De Venancio et al., in which the masseter, temporalis, occipitalis, and trapezius were injected [29]. Finally some authors state that only one muscle was injected, for example, the infraspinatus [33] or piriformis [39, 42]. Greater detail of the variability may be observed in the case of low back pain; in some studies the lumbar paravertebral muscles were injected [41], specifically the erector spinae [67], whereas other authors recommended the injection of deeper muscles, such as quadratus lumborum, psoas, and piriformis [27, 40, 43].

Such discrepancies in the muscles injected for each pain topography demonstrate either a difference between the different samples or else a difference between authors regarding the muscles considered to be the cause of each patient's pain.

Furthermore, it would probably be difficult to compare the results of studies that injected four different muscles with those that injected only one muscle. The lack of detail about the muscles injected does not help in the interpretation of the data obtained.

There is also the possibility that the treatments could be useful in a specific muscle but not in another. This idea is based on the fact that the studies performed on piriformis syndrome have reported the superiority of BTA while studies performed on another single muscle, infraspinatus [33], have not demonstrated this superiority; this leaves us with a possible source of variability according to the muscle treated.

For physicians familiarised with MPS, the selection of the target muscles for therapy is crucial. Identification of the most important active MTrPs and of other MTrPs in synergic or antagonistic muscles is a determining factor for obtaining satisfactory clinical results [45].

In summary, the marked differences in the selection of the muscle or muscles to be injected constitute another source of variability that could explain the differences in the results between trials.

### 3.3. Injection Procedure

The myofascial injection procedure is different from any other type of injection, as it requires, insofar as is possible, injection into the nucleus of the trigger point. It means that the needle will be inserted in part of the taut band that is the hardest and most tender, and that gives the most prominent twitch response. A number of techniques have been described to confirm that the injection enters the MTrP: the recommendable clinical procedure requires the LTR to be reproduced on piercing the MTrP. Hong demonstrated that the efficacy of the injection is greater when this response is obtained [68]. The use of a needle connected to an EMG device that enables the SEA to be observed can also be used to confirm that the MTrP has been reached [6]. EMG is seldom done because it is time consuming and costly. Finally, when neither of these methods is available, it is accepted that the needle is close to the MTrP if the corresponding pattern of referred pain is reproduced during the injection.

On this basis, authors have referred to injections “into trigger point” and “nearby trigger point,” that is, into the nucleus of the MTrP or close to the MTrP [52]. A third method is used in the muscles in which palpation is less reliable. For example, the psoas is reached using interventional procedures under imaging control, without taking into account where the specific MTrP is located [43].

This is a crucial issue, as the myofascial injection is specific and, as far as possible, must be performed in accordance with the standard procedure described that guarantees closest approximation to the MTrP. However, the majority of studies do not give details of the injection procedure employed and simply state “injection into the MTrP.” Some give details of the depth reached with the needle (between 1 and 3 cm) [34]. Others state that the injection was performed using the myofascial technique [29]. Finally, in two studies, it was stated that EMG control was used to locate the MTrP and perform the injection [33, 35]. Some studies have used patterns of fixed points for administration of the toxin, following a grid pattern drawn on the back [44].

Two other very important matters are the *number of trigger points* injected and the *dose of toxin* used. A very wide range of doses and of numbers of injections is reported. With onabotulinumtoxinA, there are studies performed with a single injection [30]. At the other extreme, there are studies with up to eight injections [36]. And in between, we have found studies with four [29–31] or five [37] injections. With abobotulinumtoxinA (Dysport) the number of injections varies between one [28] and 10 [34]. There are also differences in the doses used. With onabotulinumtoxinA, between five and 50 units have been used per injection site. The combination of the dose per injection and the number of points injected give a total dose that varies between 35 U [35] and 250 U [37]. With abobotulinumtoxinA the range was between 25 U [28] and 400 U [34]. The only trial designed to compare different doses of BTA was the one by Ferrante et al., in which onabotulinumtoxinA was used at doses of 10 U, 25 U, and 50 U per MTrP. No dose-dependent effect was observed [37].

There were also differences in the *dilutions* used in the preparation of the medication. OnabotulinumtoxinA is usually used at dilutions of 100 U in 1 to 2 mL, although solution volumes between 0.5 mL [33] and 10 mL [43] have been reported.

The variability in these three aspects—the injection procedure, the number of trigger points injected, and the dose per injection point or total dose—makes it very difficult to interpret the data in a unified manner. Citing once again the two largest trials, there were marked differences between treatments, one with 5 injection sites and a total dose of 250 U of onabotulinumtoxinA [37] and the other with 10 injection sites and a total dose of 400 U of abobotulinumtoxinA [34].

Another procedure-related factor is the *size of the needle* used. Although this might appear less relevant, it has been reported that the results of myofascial injection with local anaesthetic may be better with 21- or 23-gauge (G) needles [69]. Not all authors provide details of the type of needle used, but variability was also observed in those studies in which this parameter was defined, with a range of diameters between 22 G and 27 G [28, 29, 36, 37].

In summary, very significant variations have been detected in the injection procedure. Only a few studies have reported using a standardised procedure to locate the MTrP. The number of MTrPs injected varied considerably, with between 1 and 10 injection sites. There was a sevenfold variation in the total dose of onabotulinumtoxinA between the studies with the lowest and highest total doses, and studies performed with abobotulinumtoxinA presented a 16-fold difference in this parameter. This dose variability makes it very difficult to compare results between trials. There have also been up to 10-fold differences in the dilutions used in the different studies. Finally, the gauge of the needles could also affect results.

### 3.4. Treatment for Control Group

The control treatment in the majority of studies has been normal saline injection. Almost all authors considered this treatment to be a placebo [34, 37]. However, there is evidence to suggest that the injection of normal saline into MTrPs is not a placebo. Over 50 years ago, Sola et al. published two large series of patients whose pain improved after the injection of normal saline into the MTrPs [70, 71]. Frost et al. performed a study that compared the effect of normal saline and of mepivacaine on MTrPs. He found that patients injected with normal saline improved sometimes even more than those injected with the local anaesthetic [72]. The author concluded that the effect of the needle could probably be sufficient to achieve relief. This effect of saline was confirmed too at the pericranial muscles and tendon insertions for common migraine pain attacks [73].

The effect of dry needling (DN) on inactivation of MPTs is now well known. Many authors have demonstrated the usefulness of DN in MPS [68, 74, 75]. However, few studies have compared DN with BTA. The results suggest that both treatments produce an improvement. In one study there were no significant differences [29], but in another the results of the toxin were superior to those of DN, though similar to the injection of lidocaine [38].

This is an important issue, as the treatments with which BTA has been compared were not placebos but active treatments. Normal saline injection, local anaesthetic injection, and dry needling are all effective procedures, and it must therefore be taken into account that comparative studies using these techniques are trials investigating the superiority of one treatment over another, they are not placebo-controlled trials; this has implications for the determination of study sample size and for the calculation of the expected differences in improvement between the experimental group and the control group.

In addition, certain biases regarding the control treatment must be taken into account. For example, a cost-benefit study that compared the efficacy of BTA versus bupivacaine demonstrated that the two treatments produced similar improvements in the pain but that treatment with the local anaesthetic was much less expensive than BTA [36]. Ignoring the matter of cost, certainly much higher for BTA than for the local anaesthetic, one aspect of patient selection in that study should be noted as it could have favoured the results for the local anaesthetic arm: one of the recruitment criteria was prior successful injection of bupivacaine into the patient's trigger points resulting in more than 50% pain reduction for at least 8 h, but not more than 1 month. As a result, patients had to have responded favourably to one of the future study treatments in order to be included in the study, and this could have favoured that arm.

Other important details that could help to explain the variability in the results of the studies are the concomitant treatments used. For example, in one study, patients in the two treatment arms, BTA and normal saline, also received treatment with amitriptyline, ibuprofen and, when necessary, propoxyphene-acetaminophen. Myofascial release techniques were also applied to all patients for the duration of the study. It is possible that the importance of these associated treatments was not taken sufficiently into account in the evaluation of the improvement achieved in the experimental and control groups [37].

In summary, control treatments considered to be placebos have actually been treatments of known efficacy. In some studies, patients who had previously responded to one of the treatments could have been selected. Finally, some trials have included pharmacological and physical treatments administered concomitantly with the experimental and control treatments and these additional treatments could have masked the improvements observed.

### 3.5. Outcome Measures

The principal outcome variable in the majority of the studies was the difference between the pain measurements before treatment and during followup. In general, a visual analogue scale (VAS) was used [28, 30, 33, 37], although one study used a four-point pain scale [34]. Also, several standardised methods for measuring quality of life were used, mainly the SF-36 [32, 37, 44].

The majority of the studies reported that BTA was not superior to the treatments with which it was compared [27, 33, 35, 37, 38]. However, there are other high-quality clinical trials in which the opposite result was obtained, and BTA was found to be superior to the control treatment [28, 34, 41, 76]. In fact, in all the studies, all patients injected, whether with BTA or with the control treatment, presented significant improvements in their pain compared to their pretreatment pain levels. However, the pain improvement with BTA was significantly superior to that of the control treatment only in the four clinical trials indicated [28, 34, 41, 76]; in the other studies, BTA was not superior.

In the study by Ferrante et al. [37], it should be noted that although the author did not detect differences in the pain measured using the VAS, there were significant differences in the Role Emotional subscale of the SF-36 and a trend towards improvement in the Vitality and Social Functioning subscales. However, Ferrante considered that this improvement was not evaluable in a context of no improvement in the pain and that it could be explained by a type I error. Notwithstanding, it is interesting to note that other authors have also observed an improvement in the SF-36, in other subscales, specifically in bodily pain and mental health [32], suggesting that the improvement in some quality of life dimensions after treatment with BTA may not necessarily be an error.

Another of the differences detected was in the *duration of follow-up*, which varied between 4 weeks [33] and 6 months [32]. On this matter, it is worth looking at the very detailed and well-conducted clinical trial by Ojala et al. [35]. The outcome measures in that trial were pain measured using a VAS and perceived improvement on a five-point scale. A crossover design was used, in which both groups received both treatments at an interval of four weeks. Thus, the first group received BTA and, four weeks later, normal saline and the second group received normal saline initially followed four weeks later by BTA. The VAS score did not differ between the two groups in either phase of the study. However, the results of the perceived improvement scale at four weeks after the first treatment, before crossover, showed a statistically significant improvement in favour of BTA. This significance disappeared at the end of the second phase, after crossover and reinjection. Data exist that suggest that the effect of BTA persists for 12 weeks. If this is true, the improvement detected after the first treatment period, attributable to BTA, could have persisted during the second 4-week period, after the injection with normal saline, masking the results of the second phase [45].

In summary, there are contradictory results in the different trials in terms of pain improvement, with some trials that do not demonstrate superiority of BTA over alternative treatments and other trials that do confirm the superiority of BTA. Improvements have also been detected in some quality of life measures; these have been considered to be possible errors, but further explanation is required. In addition, there was considerable variation in the times at which the outcomes were measured and this may not have been ideal based on our knowledge of the duration of action of BTA.

The contradictory results in terms of superiority or nonsuperiority of BTA in the different clinical trials are the main source of doubt regarding the true efficacy of BTA in MPS associated with neck and back pain.

## 4. Systematic Reviews

The use of BT for the treatment of pain of myofascial origin has been analysed in specific systematic reviews and also in joint reviews on the usefulness of BTA in pain [45–59]. As these reviews are based on the clinical trials we have described, they present similarly contradictory results. One of the problems of the reviews could be that the results of studies are considered together regardless of the different approaches and methods used. It has been reported above that these differences are known sources of variability in outcomes and this may be one reason that the reviews provide divergent results and do not provide useful guidance for clinical practice.

The conclusions of the reviewers can be grouped into three types: BTA not recommended, a lack of data to be able to recommend or not recommend the treatment, and recommendation for use in specific conditions.

Some reviews concluded that BTA is not superior to other injection therapies, such as saline or local anaesthetic injection, and that current evidence therefore did not support the use of BTA injection into MTrPs for myofascial pain in general [50, 57] or in cervical [54] or lumbar [55] pain. Those reviewers specifically recommended not using BTA for the treatment of MPS.

Another group of reviewers concluded that the available data were not sufficiently strong either to recommend or to reject the use of botulinum toxin in MPS [51, 59]. In another review, this conclusion was expressed differently, stating that the efficacy remains unproven [56]. Finally, one review maintained both statements in its summary; that is, that there was evidence both for and against its use in myofascial pain syndrome [77]. Recommendations are usually cautious and make reference to suggestions of possible usefulness with some improvement in pain intensity and in the daily duration of pain but with more side effects with botulinum toxin. However these findings provide inconclusive evidence to support the use of botulinumtoxin in the treatment of MPS [46].

Finally, another group of reviewers concluded that botulinum toxin can be useful in MPS in certain clinical conditions.

The first of these involves pain topography. Based on high-quality clinical trials on the treatment of the lumbar pain using the toxin [41], several reviewers recommend the injection of botulinum toxin for the treatment of chronic lumbar pain [47, 56, 57] and piriformis syndrome [48, 56]. In the case of cervical pain, there are also reviews that have concluded that botulinum toxin is probably effective [48].

Another of the clinical situations is the treatment risk of the patient, for example, when the analgesic regimen carries a high potential for adverse effects [49].

One further important aspect is the difference between chronic pain and refractory pain. Refractory pain refers to pain that does not respond to other treatments. There are clinical trials and reviews that have focused exclusively on refractory pain, that is, on chronic pain that has not responded to other treatments [56, 76].

In summary, the different systematic reviews on the use of botulinum toxin in MPS and in regional axial pain (cervical, lumbar and pelvic) associated with this diagnosis, vary from no recommendation for use, through the absence of a recommendation in favour or against, or finally, to use only in specific conditions: pain refractory to treatment, and pain at specific sites (cervical, lumbar, and pelvic).

## 5. Conclusions

The use of botulinum toxin in MPS has a pharmacological and pathophysiological basis. In MTrPs there is excessive Ach release and an increase in the concentration of nociceptive neurotransmitters in the biochemical milieu of the MTrPs. BTA appears to be effective on both targets, reducing ACh release and blocking nociceptive neurotransmission.

This rational basis has been the justification for a number of clinical trials, but there are marked discrepancies between the results of those trials. Looking at the most important ones, some do not demonstrate the superiority of BTA over other treatments [27, 33, 35, 37, 38] whereas others did find differences in favour of BTA [28, 34, 41, 76].

In this paper, content analysis has been used to scrutinise the trials from a clinical perspective in order to identify sources of variability that could explain the differences in the results. The most significant findings were the following.

- Diagnostic selection and criteria: the trials have used different diagnostic criteria for MPS. There is insufficient standardisation of the different topographies of pain, with studies that have focused on a single area of the vertebral column and others that include several areas. In addition, there are very relevant discrepancies in the number of MTrPs treated and a lack of information on clinical characteristics, such as the type of myofascial pain and its associated abnormalities. The selection criteria for some studies would have led to the exclusion of their patients from other studies [34, 37].
- Muscles injected: in general, the different studies do not coincide in the target muscles. Even in patients with the same pain topography, different muscles were injected. And in some studies, the muscles treated were not even mentioned.
- Injection procedure, number of trigger points injected, and dose used: in many studies, it was not stated whether a myofascial type injection procedure was used, with robust criteria for injection into or nearby the MTrP. The number of trigger points treated showed little uniformity, as between 1 and 10 were injected, depending on the study. The doses used varied by up to 16-fold between the trials with the highest and lowest total doses. There was also variability in other factors, such as the dilution and the type of needle used.
- Control group treatments: the groups treated with placebo received treatments of known efficacy, such as normal saline, local anaesthetics, or dry needling. The investigations were therefore comparative studies between two treatments rather than an experimental group versus placebo.
- Outcome measures: the results of the trials are contradictory. In some, superiority of BTA over other treatments was not observed whereas others reported the superiority of BTA. Improvements were also detected in some quality of life measurements, and these require further explanation. Finally, the length of followup was often suboptimal if the duration of the effect of botulinum toxin is taken into account.

These marked differences have led reviewers to reach contradictory conclusions: BTA not recommended, neither recommended nor rejected or, finally, recommended for use in specific conditions of refractory pain or for pain with a specific topographic diagnosis.

### 5.1. Recommendations: to Inject or Not to Inject

The contradictory results in terms of superiority or nonsuperiority of BTA in the different clinical trials are the main source of doubt about the true efficacy of BTA in myofascial pain syndrome associated with neck and back pain.

In reality, no study has demonstrated that BTA does not improve a patient's pain. In all of them, the pre- and post-treatment outcome measures showed significant improvements. What has not been possible to demonstrate in some studies is the superiority (or inferiority) of BTA versus other treatments.

To resolve these issues, further studies must be performed that take into account these and other sources of variability described in the literature [45, 46]. The studies must apply strict criteria for the diagnosis of MPS and must evaluate the basic clinical parameters of the MTrPs. Strategies for a uniform injection technique (fixed sites) or techniques based on the patient's symptoms (follow the pain) could be established in order to identify the best approach for research. The injection procedure should be strictly myofascial and should include the use of systems available to confirm that the injection has been made into, or at least nearby, the MTrP (stimulation of LTR, SEA detection on EMG or the induction of referred pain in active points). When calculating the sample size, it must be taken into account that control treatments with local anaesthetic or normal saline are not placebos, or else a true placebo must be designed for the trials. The duration of followup for the outcome must be optimised and should probably continue for between 3 and 6 months.

In addition, in the light of all these findings, it must be concluded that there are insufficient data either to recommend or reject treatment with BTA in MPS. Many reviews close with this statement after an extensive analysis and the use of complex statistical methods, and physicians who study those reviews may therefore be left with an uncomfortable feeling that their reading has not helped to resolve the complex task of clinical decision taking.

Although this is a particularly controversial subject, some additional arguments may perhaps be given. At the present time, the treatment of MPS with BTA is an off-label indication. Some trials conclude that BTA is neither superior nor inferior to other treatments injected into the MTrPs, whereas other trials show that BTA is superior to other injection treatments. There is evidence that BTA could be useful in at least some subgroups of patients in which the most relevant characteristic is therapeutic refractoriness [56]. Some specific myofascial topographies, such as piriformis syndrome, may show a more favourable response [39]. Following this line of discussion, and complying with local regulatory procedures for off-label use, it may not be unjustified to offer this treatment to patients with refractory MPS that has not responded to other forms of injection therapy. If it is decided to use this approach, all these details must be explained to the patient by means of an informed consent. Under these conditions, the feeling of some physicians is that treatment with BTA may be helpful to the group of patients that does not respond to other myofascial treatments.
